# *In silico* approach to predict pancreatic β-cells classically secreted proteins

**DOI:** 10.1042/BSR20193708

**Published:** 2020-02-14

**Authors:** Erika Pinheiro-Machado, Tatiana Orli Milkewitz Sandberg, Celina PIHL, Per Mårten Hägglund, Michal Tomasz Marzec

**Affiliations:** 1 Department of Pathology and Medical Biology, University Medical Center Groningen, University of Groningen, The Netherlands; 2 Department of Biomedical Sciences, University of Copenhagen, Denmark; 3 Tel Aviv University, Israel

**Keywords:** Beta cells, pancreatic islets, protein secretion, secretome

## Abstract

Pancreatic β-cells, residents of the islets of Langerhans, are the unique insulin-producers in the body. Their physiology is a topic of intensive studies aiming to understand the biology of insulin production and its role in diabetes pathology. However, investigations about these cells’ subset of secreted proteins, the secretome, are surprisingly scarce and a list describing islet/β-cell secretome upon glucose-stimulation is not yet available. *In silico* predictions of secretomes are an interesting approach that can be employed to forecast proteins likely to be secreted. In this context, using the rationale behind classical secretion of proteins through the secretory pathway, a Python tool capable of predicting classically secreted proteins was developed. This tool was applied to different available proteomic data (human and rodent islets, isolated β-cells, β-cell secretory granules, and β-cells supernatant), filtering them in order to selectively list only classically secreted proteins. The method presented here can retrieve, organize, search and filter proteomic lists using UniProtKB as a central database. It provides analysis by overlaying different sets of information, filtering out potential contaminants and clustering the identified proteins into functional groups. A range of 70–92% of the original proteomes analyzed was reduced generating predicted secretomes. Islet and β-cell signal peptide-containing proteins, and endoplasmic reticulum-resident proteins were identified and quantified. From the predicted secretomes, exemplary conservational patterns were inferred, as well as the signaling pathways enriched within them. Such a technique proves to be an effective approach to reduce the horizon of plausible targets for drug development or biomarkers identification.

## Introduction

The term secretome was first defined as the whole subset of factors secreted by a cell [1] and later revised to ‘all proteins secreted by the cell into the extracellular space’ [2]. These factors can regulate a multitude of physiological processes and dictate the composition of the extracellular environment. Secretome analysis in health and disease can, therefore, bring insights about the pathophysiology of these conditions, opening new perspectives for the discovery of biomarkers and therapies [3].

Classically, proteins are secreted through the secretory pathway [4]. mRNAs are processed by ribosomes in the cytoplasm and the proteins containing a signal peptide (SP) – motif often composed by one positively charged amino acid followed by 6–12 hydrophobic amino acids [5] – are cotranslationally translocated to the endoplasmic reticulum (ER) [6]. Once there, proteins are folded by chaperones and foldases to gain proper 3D conformation [7]. The ER has its own subset of native proteins, the so-called ER-resident proteins [8]. Their continuous localization to the ER lumen is secured by an ER-retrieval signal located in the C-terminal part of the protein [8]. This signal is commonly a tetrapeptide – K (lysine/lys) D (aspartic acid/asp) E (glutamic acid/glu) L (leucine/leu) – or related sequences [8,9] and enables proteins to bind to KDEL-receptors at the early Golgi, thus preventing proteins from being secreted from cells [10].

The non-ER-resident proteins are forwarded to the Golgi complex where different post-translational modifications occur [11]. Typically, proteins that are ready to be secreted are sorted into vesicles that: (1) can undergo secretion (exocytosis) [12]; (2) can wait for signals to undergo exocytosis [13]; or (3) can be translocated to the lysosomes for degradation [14,15].

Proteins can be secreted via conventional and unconventional secretory pathways [16,17]. The conventional secretory pathway is responsible for transport and secretion of proteins containing SP, while the mechanisms behind unconventional secretion remain elusive [18–20]. Around 39% (7649) of all human protein-coding genes (19,613) are predicted to display either SP and/or transmembrane regions [21]. The number of potentially secreted proteins (with SP and no transmembrane regions) were described to be 2623, which accounts for approximately 13% of all proteins [21,22]. Unconventionally secreted proteins often lack SP, are not localized to the secretory pathway organelles, and their secretion continues even in the presence of brefeldin A (an inhibitor of ER-Golgi transport) [23,24], imposing another layer of complexity in the secretomes’ characterization.

Several approaches to define a secretome of a given cell type were employed in the past, but the introduction of proteomics greatly facilitated the overall methodology [20,25,26]. Initially, investigated with the use of 2D gel electrophoresis, radiolabeling, and enzyme activity [27,28], the secretomes were described with only a few components. Currently, at the advent of better techniques in bioinformatics and mass spectrometry (MS), the identification of hundreds to thousands of proteins is becoming routine and such approaches were successfully implemented in the description of secretomes [29–31]. However, a simple and user-friendly tool that systematically integrates experimental high-throughput proteomic datasets using proteomic data from any kind of cell type is still missing.

**Figure 1:**
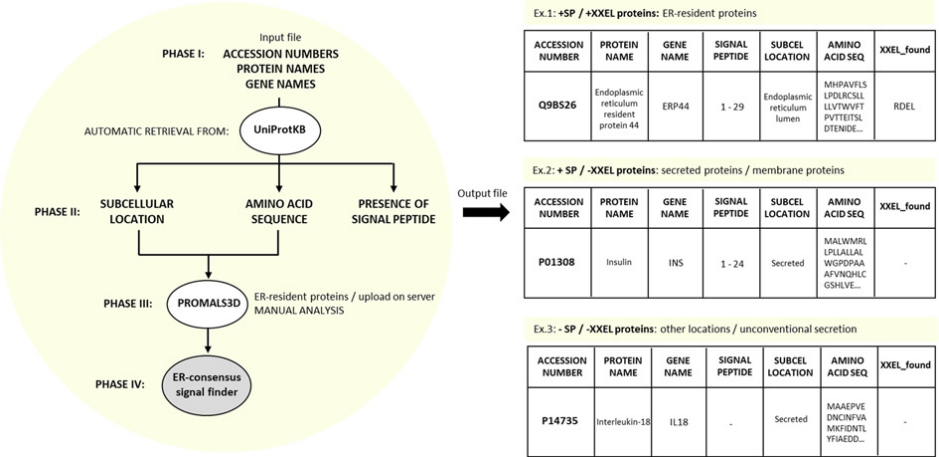
The applied method workflow The script processes phases II and IV automatically. Phase I is done manually by the user, organizing the input file according to what is required: accession numbers, protein and gene names. Phase II (retrieval) is automatic, and the data is retrieved from UniProtKB. Phase III relies on the PROMALS3D server, in which the input data need to be manually organized and submitted. Phase IV is based on the PROMALS3D alignment results, where the ER-retrieval signal consensus is searched through all proteins in the list automatically. Examples of possible output data shown.

In the present study, a computational tool for the prediction of classically secreted proteins that only requires a list of identified proteins was developed (Figure 1). An open source Python tool that rationally integrates public databases, facilitates the analysis of protein lists, and that can be modified according to the cell type of interest, has been designed. Our method makes possible to retrieve, organize, search and filter data regarding the full amino acid sequence, protein subcellular location, presence of SP, and presence of a consensus of ER-retrieval signal (Figure 1). The approach was utilized in the analysis of available datasets from human pancreatic islets (HPI) [32], mouse pancreatic islets (MPI) [33], isolated β-cells [32], rat insulin secretory granules (ISG) [34], β-cells supernatant (SUP) [35] as well as our own supernatant of β-cell model cells, the insulinoma INS-1E, upon high glucose stimulation (SUP2) (Table 1). The tool predicted the classically secreted proteins by enabling clustering of proteins containing SP (+SP) and ER-resident proteins, which were later used to filter out a range of 70–90.5% of the original data analyzed. From this predicted subsets, conservational functional patterns were inferred, as well as the signaling pathway enriched in them.

**Table 1:** Subset of all publications that fulfill the following criteria: publication within the last 10 years; list of proteins identified available as an excel file. Publications chosen to validate our Python tool are highlighted in light yellow. N/A: non-applicable, i.e. cells were not submitted to any special experimental conditions

	Study / Author, year	Species	Condition	Technique	# Identified Proteins	Ref.
**Whole-cell**	Glucose-stimulated islet proteome / **Waanders et al. (2009)**	Mice (islets)	High glucose	LC-MS/MS (LTQ-Orbitrap)	6902	[39]
	Glucose-stimulated mice β-cell proteome / **Martens et al*.* (2010)**	INS-1E	High glucose	Alternate scanning LC-MS	300	[33]
	The Human Diabetes Proteome Project / **Topf et al*.* (2013)**	Human (islets)	N/A	Gas-Phase Fractionation MS	5317	[32]
	The Human Diabetes Proteome Project / **Topf et al*.* (2013)**	INS-1E	N/A	LC-MS/MS	2625	[32]
**β-Cell secretory granule**	Insulin granule / **Brunner et al*.* (2007)**	INS-1E	N/A	Granule purification, LC-MS/MS	130	[40]
	Insulin granule / **Schvartz et al*.* (2012)**	INS-1E	N/A	SILAC, 3-step gradient purification, MS/MS	140	[34]
	Insulin granule / **Li et al*.* (2018)**	INS-1	N/A	OptiPrep, (LC)–MS/MS, correlation profiling	81	[41]
**β-Cell supernatant**	β-Cell secretome / **Tattikota et al*.* (2013)**	MIN6 cells supernatant	High Glucose	Concentration (3MWKO), EASY-nLC MS/MS	1629	[42]
	β-Cell secretome / **Pepaj et al*.* (2016)**	INS-1E cells supernatant	Vitamin D exposure	SILAC, LC-MS/MS	821	[35]

## Research design and methods

### Chemicals and materials

All reagents were purchased from Sigma, Søborg, Denmark. Additionally, trypsin from porcine pancreas (Promega, Madison, Wisconsin, U.S.A.); 1,4-dithiothreitol (DTT), iodoacetamide (IAM), urea 98%, Tris base ≥99.9%, trifluoroacetic acid (TFA), acetonitrile grade liquid chromatography-mass spectrometry (LC-MS) Chromasolv, and water-grade LC-MS Chromasolv (Thermo Fischer Scientific, Hvidovre, Denmark) were used. Vivacon 500 Ultra centrifugal filters with a 2-kDa molecular weight cut-off (2 MWCO) were purchased from Sartorius Stedim Lab Ltd. (Stonehouse, Gloucestershire, U.K.).

### Cell culture

The rat insulinoma INS-1E cell line was grown in RPMI-1640 GlutaMAX medium (11 mM glucose) supplemented with 10% fetal bovine serum (FBS), 100 U/ml penicillin, 100 μg/ml streptomycin, 10 mM HEPES, 1 mM sodium pyruvate and 50 μmol/l β-mercaptoethanol.

The cells were grown in 6-well plates until 85–90% confluence. Each well received fresh medium 12 h prior to the experiment. Due to reduced efficiency of MS analysis in the presence of FBS, each well was washed 3× with RPMI-1640 glucose and FBS free, supplemented with 100 U/ml penicillin, 100 μg/ml streptomycin, 10 mM HEPES, 1 mM sodium pyruvate and 50 μmol/l β-mercaptoethanol. After washing, cells were incubated with RPMI-1640 FBS-free 20 mM glucose (2 ml) for 4 h prior to the collection of the supernatants. Cell numbers used in experiments: 3 × 10^6^ INS-1E cells (or the equivalent of 85–90% confluence of a 6-well plate).

### Sample preparation and protein digestion

After the incubation, conditioned media from INS-1E cells were collected and centrifuged (2000 ***g***, 10 min) to remove cell debris. Samples were concentrated using Vivacon 500 2 MWCO filter units (Sartorius, Goettingen, Germany) followed by an adapted protocol from Wisniewski et al*.* [36]. Proteins in the filter were reduced in a denaturing buffer (8 M urea, 0.1 M Tris-HCl pH 8.5, 500 mM DTT) for 30 min at room temperature (RT) and followed by alkylation of free sulfhydryl groups with 500 mM IAM at RT for 30 min in the dark. Reduced and alkylated samples were incubated overnight with trypsin from porcine pancreas. Next day, samples were centrifuged and the flow through, where the peptides were contained, collected.

### StageTips preparation

For micro-purification of peptides prior to MS, an adaptation of Rappsilber et al*.* [37] was used. The StageTips were prepared placing two Empore filter disks (3M) at the very end of a D200 200 μl tip using a sampling tool syringe. Each sample had the pH adjusted to approximately 2 with 10% TFA. After filter activation, the samples were loaded and centrifuged (1200 ***g***, 3 min). The StageTips were washed with 0.1% TFA and peptides were finally eluted with 60% acetonitrile, 0.1% TFA. Acetonitrile was evaporated at RT prior to MS analysis.

### Mass spectrometry

Samples were analyzed on a Bruker Impact II ESI-QTOF (Bruker Daltonics, Bremen, Germany) mass spectrometer with an on-line Dionex Ultimate 3000 chromatography system (Thermo Fisher Scientific, Waltham, Massachusetts, U.S.A.) equipped with a Bruker Nanoelute column (15 cm, 75 µm ID). Peptides were eluted using a solvent gradient over 65 min, using acetonitrile with 0.1% formic acid at a flow rate of 300 nl min^−1^. The MS scan range was 150–2200 *m/z* with a cycle time of 3 min using a MS sampling rate of 2 Hz followed by intensity-based data-dependent MS/MS (4–16 Hz).

### Data analysis

Database searches were performed with MaxQuant v 1.6.1.0 [38] using the following parameters: enzyme: trypsin, with three missed cleavages; fixed modification: carbamidomethyl (cysteine); variable modification: oxidation (methionine); 1% peptide-level false discovery rate; mass tolerance: 0.07 and 0.005 Daltons (first and main searches, respectively); MS/MS mass tolerance: 40 part per million (ppm) (first and main searches). Data analysis was performed using the same software (MaxQuant v 1.6.1.0) [38] with semi-specific tryptic constraints and a 1% peptide level false discovery rate. Proteins identified by at least one unique peptide were considered only if their presence was consistent in two out of the three samples submitted for analysis. Further, the list was additionally filtered to exclude potential contaminants.

### Proteomic datasets analyzed

An overview of the publications relevant to our analysis is presented in Table 1. The publications from which the lists of proteins were acquired: (1) HPI [32], (2) MPI [39], (3) INS-1E cells (isolated β-cells) [32], (4) ISG from INS-1E cells [34], and (5) INS-1E cell supernatant (β-cells SUP) [35], were extracted and then analyzed by our tool and are highlighted in Table 1. All lists are public and available. Additional lists were found but not analyzed [33,40–42]. The sample processing and proteomic approaches vary between datasets and their descriptions can be found in respective publications. Our own list of proteins from the supernatant of INS-1E cells after 20 mM glucose stimulation, 4 h (β-cell supernatant – SUP2) presents 978 proteins, and it was additionally used as a proteomic dataset in the present study.

### Components of the analysis method

The method is divided into four phases (Figure 1).

#### Phase I: the assembly of the data (manual)

Outputs from proteomic studies are typically presented as excel files containing a list of the identified proteins. From the lists available in the chosen publications, the identified protein names and their respective gene names and accession numbers – unique stable identifiers assigned to each protein by the UniProt Knowledge (UniProtKB, v. 2019_03 updated in April 2019) database [43] – were extracted and copied to a new excel file. All subsets were submitted to the same handling for file organization. Consequently, a readable file for our tool is an excel file containing: (1) accession numbers, (2) protein names and (3) gene names.

#### Phase II: retrieving the subcellular location, full amino acid sequence and SP (automatic)

After assembly of the input file during phase I, the data from UniProtKB regarding each protein’s subcellular location, as well as their full amino acid sequence and the presence of SP are retrieved. All proteomic datasets were submitted at least three times to check for potential inconsistencies in the data retrieval. Obsolete and deleted entries that were identified after UniProt retrieval were excluded from the respective datasets. The output file after retrieval is an excel file containing the features retrieved in separated columns. Within an excel file, the auto filter feature was employed. It facilitates the selection of +SP proteins or the ones containing an ER-retrieval consensus. The auto filter also facilitates the screening of subcellular locations (once selected only “secreted”, the excel will show only proteins annotated to be secreted according to UniProtKB). Details about the script can be found in the documentation (Supplementary File S1).

#### Phase III: ER-retrieval signal consensus (manual/automatic)

Once the subcellular locations are displayed in the excel file, it is possible to filter the proteins that were experimentally proven to localize to the ER by again employing the excel auto filter. This filtering was made using the HPI list, the largest subset of proteins we had access to, and where we could find the largest number of ER-resident proteins. The list submitted to PROMALS3D is available in the Supplementary File S2. With this subset of human islet ER-proteins and their full amino acid sequence, a server for amino acid sequence alignment was used. PROMALS3D (PROfile Multiple Alignment with predicted Local Structures and 3D constraints) is a tool for multiple sequence and structure alignment [44]. The server receives input sequences in a FASTA format and it is able to return a consensus (the most common tetrapeptide variation within human islet ER-resident proteins, Supplementary File S3).

#### Phase IV: ER-consensus finder (automatic)

The final step of the script executes the search of the consensus in the four last amino acids from the full amino acid sequence of each protein in the original list. All lists were submitted at least three times to perform this search. All proteins in all lists have their amino acid sequences described and appropriately recovered in phase II. If the consensus is found in the C-terminus, it will return the information in a new column in the outcome file.

The objective function of our approach can be summarized as:

Classically secreted proteins = (x2 – x3)

The objective function was implemented in our script as:

# Second, Grab the raw protein sequence
sequence = soup.select(‘sequence[length]’)[0].get_text().replace(‘\n’, ”)
excel_new[’full_sequence’][i] = sequence
# If the protein has a signal peptide, parse the information
features = soup.select(‘feature[type = “signal peptide”]’)
*if len(features) > 0:*
  # Grab start and end position of the signal peptide
  feature = features[0]
  start = feature.select(‘begin’)[0].get(‘position’) # Get start position
  end = feature.select(‘end’)[0].get(‘position’) # Get end position
  excel_new[‘signal_sequence’][i] = start+” ”+end
  # Last, look for the KDEL in the final 4 amino acids
  searcher = re.compile(‘[ARNDCQEGHILKMFPSTWYVBZ]{2}EL’)
  # Select last four characters of the full sequence
  final_aa = sequence[-4:]
  # Check if there is a match with the regular expression
  matched = searcher.match(final_aa)
  *if matched:*
    excel_new[‘kdel_found’][i] = matched.group(0)

## Results

The semi-automated method that integrates relevant functionalities for the prediction of classically secreted proteins (classical secretome) was constructed. The different components of the workflow were linked using a Python script. All datasets, including our own, were submitted for analysis by our tool, and the results are described and discussed below.

### Data selection and organization of the readable file

A literature review was conducted in order to identify data sets of the pancreatic β-cell secretome upon high glucose stimulation, presented as a straightforward list that could be used as a reference. To the best of our knowledge, this list is not available. Therefore, a new analysis was carried out. As a source of datasets and a proof of principle, nine specific publications containing proteomic data from HPI, MPI, β-cells, ISG, and SUP were selected (Table 1). By applying the criteria: (1) publication date (data reported in the last 10 years) and (2) the full list of identified proteins available as supplementary material in the original publication (excel file), five out of the nine publications were selected for further analysis (Table 1 – highlighted publications). The resulting list from our own analysis of the INS-1E cells upon 20 mM glucose stimulation (SUP2) was also submitted.

### Filtering by the presence of SP – potential structure for secreted proteins

Among the 5317 proteins identified in the HPI proteome [32], 13.6% of proteins display SP (+SP) and therefore potentially belong to the classical secretory pathway. Relatively proportional rates were found in MPI (10.5%) and β-cells (10%). The ISG, as a structure that undergoes exocytosis, was expected to contain only proteins that went through the secretory pathway, i.e. +SP. Indeed, it was the dataset containing the highest percentage of +SP proteins (30.7%). Filtering the list from the β-cell SUP as well as SUP2, both presumed to be highly enriched in secreted proteins, the percentage of +SP proteins were only 13 and 9.4% respectively. Detailed descriptions are presented in Table 2.

**Table 2:** The summary of performed analysis. SP: signal peptide; XXEL: KDEL signal consensus. +SP - XXEL: predicted secretome (classical secretome)

	Study / Author, year	Proteins analyzed	Signal Peptide SP	ER-resident proteins +SP +XXEL	Predicted secretome +SP -XXEL
Human islet (HIP)	The Human Diabetes Proteome Project / Topf et al*.* (2013)	5317	725 (13.6%)	53 (1%)	672 (12.6%)
Mice islet (MIP)	Glucose-stimulated islet proteome / Waanders et al*.* (2009)	6745	708 (10.5%)	59 (0.9%)	649 (9.6%)
INS-1E Rat β-cell (β cell)	The Human Diabetes Proteome Project / Topf et al*.* (2013)	2523	254 (10%)	17 (0.67%)	237 (9.4%)
INS-1E ISG (ISG)	Insulin granule / Schvartz et al*.* (2012)	140	43 (30.7%)	1 (0.7%)	42 (30%)
INS-1E supernatant (SUP)	β-cell secretome / Pepaj et al*.* (2016)	823	104 (12.6%)	13 (1.6%)	91 (11%)
INS-1E supernatant (SUP2)	β-cell secretome / our subset	978	92 (9.4%)	13 (1.3%)	79 (8%)

### ER-consensus sequence – identification of a functional tetrapeptide for pancreatic islets

A continuous retrograde transport of ER-resident proteins from the cis-Golgi is necessary to retrieve the resident soluble proteins to the ER lumen [8,10]. The selection of these proteins is dependent on a tetrapeptide present in the C-terminus, classically referred to as KDEL motif in animal cells and HDEL in *Saccharomyces cerevisiae* [8,9]. However, multiple functional variants of this motif have been reported, with the main restriction that the last two amino acids are glu-leu (EL) [45]. The process of identifying the human islets ER-resident proteins was already described in the methods session (phase III/Supplementary Files S2 and S3) and indeed the most common motif identified was EL at the last two C-terminal amino acid positions. In agreement with previous publications, high variability at two preceding positions was identified with amino acids H, S, R, P, E, F, G and D substituting K at first position of KDEL motif and T, N, E, V, G, A, I and R substituting D at the second position. Considering that protein list submitted for this analysis was biased to the experimental approaches that led in the first place to their identification as ER luminal residents, and to maintain the possibility that other ER-localized proteins with different KDEL-like motifs are not filtered out during unbiased proteome–secretome analyses, we decided to use only EL sequence for further filtering. However, as KDEL motif interaction with KDEL receptors is impacted by four C-terminal amino acids and keeping with the traditional annotation, we have further referred to the motif as XXEL, where X represents any of the 20 possible amino acids found in living organisms.

Our analysis showed that 1% of the HPI (53 proteins), 0.9% of the MPI (59 proteins) and 0.67% of β-cells (17 proteins) proteomes contain the motif XXEL (+XXEL). The ISG displayed only 1 protein with the XXEL motif, the endoplasmic reticulum chaperone BiP while the supernatants (SUP and SUP2) presented the largest amounts (proportionally): 1.6 and 1.3% (13 proteins both).

### The predicted subset of classically secreted proteins: classic secretomes

The final goal of our approach was to predict, *in silico*, the classically secreted proteins. Although being aware that the secretome contains ‘all factors secreted by a cell to the extracellular space’ [1,2], we will here name the subset of classically secreted proteins as the ‘classical secretome’. All phases of the program converged to identify proteins that are concomitantly +SP and -XXEL, thus generating a list that fulfills the known structural requirements necessary for secretion through the classical secretory pathway. The auto filter feature from excel was also used within the ‘subcellular location’ column (phase II, previously described). The auto filter provides another layer of refinement, allowing the search for specific groups of locations. Additionally, we filtered the ISG and supernatants (SUP, SUP2) data to obtain a ‘cleaner’ list, excluding intracellular contaminants or proteins outside of the +SP -XXEL criteria.

Following this reasoning, the tool reduced the proteomes to the *in silico* predicted secretome – classical secretome (Table 2/Supplementary File S4). HPI, MPI, β-cells, ISG, SUP and SUP2 were reduced, respectively, by 87.4, 90.4, 90.5, 70, 88.8 and 92% of their full identified proteome. In other words, 12.6 (HPI), 9.6 (MPI) and 9.5% (β-cell) of proteins met the criteria to be included in the final list of respective, classical secretomes. Surprisingly, only 30% of the proteins identified in the ISG were +SP -XXEL proteins. Regarding the analyzed supernatants only 11% (SUP) and 8% (SUP2) fulfilled our criteria of a classical secretome (Supplemenatry File S5).

### Comparative analysis – conservation among the predicted secretomes

**Figure 2:**
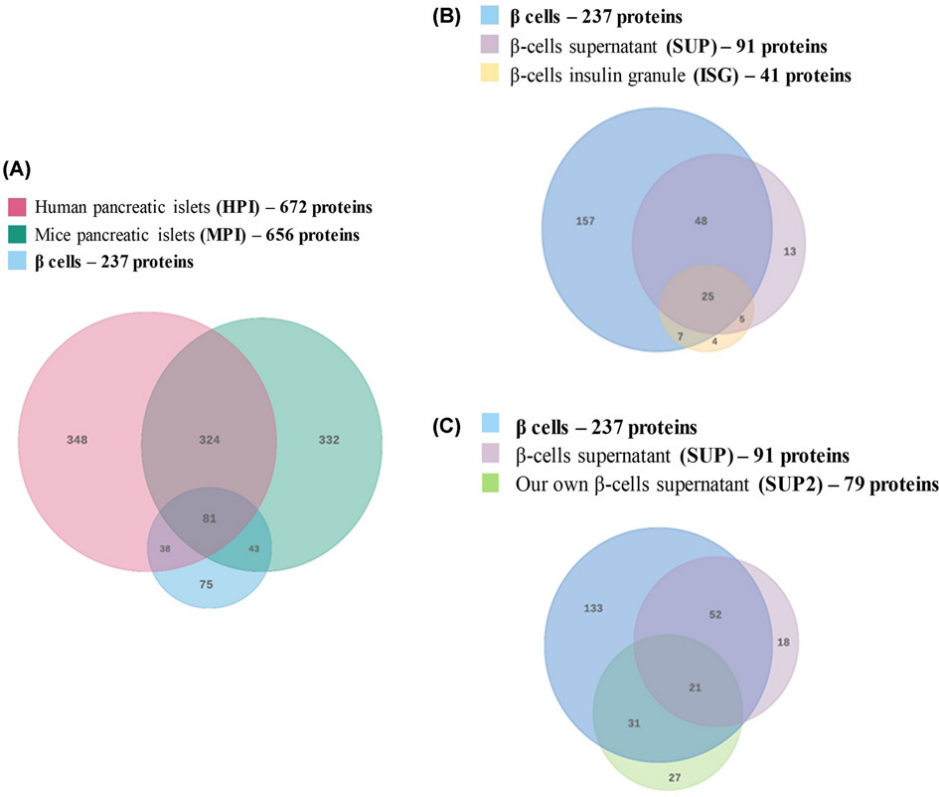
Venn diagrams showing overlaps between different subsets of classically secreted proteins (secretomes) (**A**) β-Cell secretome and mice pancreatic islets (MPI) are approximately 50% similar to human pancreatic islets (HPI). (**B**) The filtered β-cell insulin granule (ISG) and the β-cell supernatant (SUP) share around 80% identity with the β-cell secretome. (**C**) β-Cell secretome (SUP2) shares 50% similarity with the β-cell supernatant (SUP). Both supernatants (SUP and SUP2) share 80.2 and 65.8%, respectively.

Although conservational studies among secretomes are not uncommon investigations [46,47], the analysis of how conserved the secretomes of human and rodent islets/β-cells are is still a loose end. Our data showed that HPI and MPI predicted secretomes are 49.4% similar. Using the β-cell predicted secretome as a reference, 119 proteins (out of 237) are enclosed within the HPI classical secretome, indicating that β-cells and HPI share 50.2% similarity in their secretomes. β-Cells and MPI classical secretomes share 52.3% identity. About 78% proteins reported in ISG where found in β-cell predicted secretome as well (32 out of 41). Finally, comparing the ISG to filtered SUP, we observed 73% similarity. Interestingly, the comparison of predicted secretomes of HPI and MPI to β-cell yielded 81 common proteins. However, when we compared limited with β-cells, predicted secretomes of SUP and ISG or SUP and SUP2, we identified only 25 and 21 common proteins. Overlapping patterns are presented in Figure 2.

### Pathways that are enriched in the human islet predicted secretome: analysis of *in silico* obtained data set

To evaluate the biological relevance of *in silico* prediction of secretome(s), we have analyzed the biggest protein subset (*in silico* HPI secretome) for the pathway over-representation. We used Reactome, an open-source, curated and peer-reviewed pathway database [48] for analysis. Based on identification of 498 out of the 672 proteins submitted, Reactome generated a list of the 25 pathways found in the human islet predicted secretome sorted by p-value (Supplementary File S6). The list of enriched pathways clustered in three groups: (1) cell adhesion/extracellular matrix (ECM) interactions, (2) immune system and coagulation, and (3) cell proliferation (Table 3).

**Table 3:** Clusters with the enriched pathways identified in the predicted human islets secretome according to Reactome database

CLUSTERS	PATHWAYS
**CELL ADHESION / ECM INTERACTIONS**	Laminin interactions
	Integrin cell surface interactions
	Extracellular matrix (ECM) proteoglycans
	Non-integrin membrane–ECM interactions
	Assembly of collagen fibrils and other multimeric structures
	Degradation of the ECM
	ECM organization
	Collagen formation
	Collagen degradation
	MET activates PTK2 signaling
	Collagen biosynthesis and modifying enzymes
	Elastic fibre formation
	MET promotes cell motility
**IMMUNE SYSTEM / COAGULATION**	Neutrophil degranulation
	Platelet degranulation
	Response to elevated platelet cytosolic Ca2+
	Formation of Fibrin Clot (Clotting cascade)
	Platelet activation, signaling and aggregation
	Innate immune system
**CELL PROLIFERATION**	Regulation of insulin-like growth factor (IGF) transport and uptake by insulin-growth factor binding proteins (IGFBPs)
	Syndecan interactions
**OTHER**	Post-translational protein phosphorilation
	Retinoid metabolism and transport
	Metabolism of fat-soluble vitamins
	Glycosphingolipid metabolism

## Discussion

We showed here the utility of a new method to analyze proteomic data. It is a straightforward approach that can be used prior to the laboratory validation process, helping to support research questions (Figure 1). In order to accomplish its functions of filtering for classically secreted proteins, we have selected UniProtKB and PROMALDS3 as the reliable database and server, as well as Python as the programming language. All components necessary for our approach to function properly are free and publicly available at *https://github.com/tatiorli/ParseSignalPeptides*. The documentation can be found in the Supplementary File S1.

UniProtKB provides a comprehensive and freely accessible resource of protein information [49]. Manually annotated and reviewed records extracted from literature and curator-evaluated computational analysis [43,49]. From UniProtKB, we retrieved data regarding subcellular location, full amino acid sequence and the presence of SP of each protein present in the input list. The subcellular location was used to increase the analysis power of our tool although this feature has to be applied cautiously as some proteins’ annotations are (1) derived experimentally while other are not thus introducing a strong bias, and (2) some proteins are shown to localize to two or more cellular compartments. The proteins’ full amino acid sequences, on the other hand, subsequently used for the XXEL detection were available for all tested proteins. The SP presence is dictated by UniProt if the cleavage site has been determined by direct protein sequencing or if it was predicted by at least two different predictive tools (Phobius, Predotar, SignalP, and TargetP). The credibility and wide usage of the database provides a strong argument to employ it as a central database for our method.

The ER-retention signal is crucial for the recovery of ER-resident proteins [10]. Classically, the process is mediated by receptors that recognize the KDEL motif, returning a protein that has escaped to the ER lumen. Three different receptor homologs were described in humans, forming the KDEL receptor family [50] and they exhibit varying affinities for KDEL motif variants [45]. So far, 59 KDEL variants have been identified experimentally [45]. We rationalized that other functional KDEL variants may exists and thus decided on an unbiased approach to identify them. We have aligned the amino acid sequences of experimentally proven ER-resident proteins (HPI data set) and identified only the last two C-terminal amino acids: EL as the consensus sequence. The first two amino acids within KDEL motif, varied among proteins (Supplementary Files S2 and S3) and in agreement with previous publications [45]. To avoid bias, where only experimentally proven ER-resident proteins are used for KDEL variant identification, we decided to use for protein filtering the most common identified EL consensus (for consistency referred to as XXEL) and not a limited number of KDEL variants found via alignment in the tested data set (Supplementary File S3). Clearly, the future experimental verification of each possible variant functionality is necessary. This approach limits false negative selection (non-classical KDEL sequence) but maintains a 14.4% level of false positive choices. It is important to point out that the current assignment of proteins to other than ER cell compartments (false positive group) may change as new experimental data is provided, lowering the rate of false positive identifications.

Further validation of our approach derives from the decrease in the numbers of XXEL proteins proportionally to the total number of identified proteins through the models. While the HPI and MPI contained 53 and 59 +XXEL proteins respectively, the β-cell presented 17 and the ISG only 1 (Table 2). The only +XXEL protein found within the ISG is the endoplasmic reticulum chaperone BiP, the ER chaperone already described in the context of the extracellular compartment [51,52]. Regarding the β-cell supernatants (SUP and SUP2), both showed 13 +XXEL proteins (Table 2), indicating probable contamination with cellular matter and/or secretion of ER proteins by the insulinoma cell model, INS-1E. According to the Human Protein Atlas, around 2% of all human proteins have been experimentally shown to localize to the ER [53]. Another report states that 202 ER-proteins are found in the pancreas ER (corresponding to around 1% of all human proteins) [54]. Our results showed proportional trends (approximately 1%) suggesting a high level of consistency between our screening and other published approaches (Table 2). If only the cellular localization filter is used to search for ER proteins, many more than the ones in the list of +XXEL proteins will be found. The probable reason lies in the fact that 50% of all ER proteins are shown to co-localize to other compartments such as cytosol and nucleus [53].

It is reported that 39% of the human protein-coding genes are predicted to have a SP [21]. Considering that the HPI proteome is one-third of the human proteome [32] and that we showed it contains 13.6% +SP islet proteins, the results are coherent. MPI and β-cells exhibit similar proportions (±10%). The ISG and supernatants (SUP, SUP2) were expected to present higher percentages since they are predicted to contain only ready-to-be secreted or already secreted proteins. Surprisingly only ISG showed substantial increase (up to 30%) while SUP and SUP2 contained only 12.6% and 9.2% of +SP proteins (Table 2). This can be explained by (1) the presence of intracellular contaminants as a result of sample processing, (2) a large number of unconventionally secreted proteins (that do not fulfill our criteria of +SP), (3) a reflection of new biological processes by which β-cells can secrete proteins e.g. formation of exosomes [55] or (4) a characteristic of insulinoma INS-1E cell line. It is important to mention that the presence of SP is not a guarantee of secretion, as +SP proteins can eventually be retained in the lumen of the ER, lysosomes, any other organelle along the secretory pathway or in the membranes (e.g. membrane bound receptors).

Our ultimate goal was the creation of a list of the islet and β-cell classically secreted proteins that we achieved by reducing the human and β-cell proteomes to +SP -XXEL proteins (Supplementary File S4). With no major differences in the proportions, HPI and MPI, as well as β-cells contain 12.6, 9.6 and 9.4% of proteins within their respective proteomes that represent what we call the classical secretome (Table 2). This secretome includes many proteins that fit our criteria (+SP -XXEL) but that are not elsewhere annotated as secreted. It includes membrane proteins and proteins from organelles in the secretory pathway (i.e. lysosomes). To further narrow down the secretome list multiple approaches can be suggested, e.g. (1) usage of the cellular localization filter, (2) immunostaining of individual proteins or (3) exogenous expression of the tagged protein of interest.

A series of reviews and reports analyze how translatable are the discoveries and therapeutic perspectives made in rodent models to humans in the field of diabetes [56]. These discussions are especially important in order to choose between models that can best serve for the purposes of clarifying the specific mechanisms behind diabetes development and progression. The considerations at the protein expression level utilize the comparisons at the whole cell proteome or transcriptome level [57]. We decided to analyze how similar the subsets of *in silico* predicted classical secretomes of HPI and β-cells are (Figure 2). After filtering made by our tool, the lists of predicted classically secreted HPI and MPI proteins were overlaid. The overlap we obtained came as a surprise: 50.2% (Figure 2). The variance may represent species differences and/or reflect technical hindrances e.g. protein annotation and naming differences between animal species, databases redundancies, obsolete and deleted accession numbers, mass spectrometry related issues (machine and software used to analyze input samples) and finally, sample preparation and experimental design.

Much higher evidence of overlap was seen when 78% of the +SP -XXEL ISG proteins matched with the *in silico* β-cell secretome, as well as the 73% matching with the +SP -XXEL SUP (Figure 2). Moreover, the predicted β-cell secretome and the +SP -XXEL from SUP share 80% identity. Interestingly, our β-cell secretome (SUP2) is only 65.8% similar to the SUP secretome (Figure 2). This difference possibly arises from the different research designs and sample processing, where SUP is a supernatant from β-cells exposed to vitamin D [35], our (SUP2) comes from β-cells after high glucose exposure (20 mM), underscoring biases related to experimental design.

Another analysis was carried out in order to support the reliability of the filtering made by our tool. Using Reactome database, the biological processes enriched in the HPI classical secretome were investigated. The 25 most relevant pathways were identified (Supplementary File S6), and we clustered them in three groups (Table 3). The first, ECM interactions and cell adhesion group, indicate an active islet participation in creation and modeling of extracellular microenvironment where different cell types need to coexist. This subject has gained a special attention in the islet transplantation field, where ECM molecules have been explored in their clinical potential to contribute to the islet’s engraftment success [58]. The second group is related to the immune system and consists of proteins related to neutrophil and platelet activation and degranulation, activation of other innate immune system components, and glycosphingolipid metabolism. These factors are tightly involved in the regulation of the inflammation levels that have been associated with the development of diabetes and islet homeostasis [59,60]. Finally, the third group was mainly represented by the insulin growth factors (IGFs) signaling and metabolism of fat-soluble vitamins. Given that the IGFs were shown to be involved in β-cell survival and insulin secretion, their presence in the extracellular space is of no surprise [61,62]. On the other hand, it is interesting to highlight that pathways involved in the fat-soluble vitamins (vitamins A, D, E, and K) metabolism are enriched in the HPI secretome. Recently, a report showed that a lack of vitamin A can lead to β-cell dysfunction and insulin production in mice [63], while vitamin D has been investigated for its potential properties to protect and repair damaged β-cells [64]. Although 782 pathways were identified by at least one of the secreted proteins on our list, no pathways regarding well known intracellular processes (DNA replication, chromatin organization) were significantly enriched, demonstrating that our analytical approach provides a good level of clearance of intracellular contaminants.

The pancreatic islets constitute 1–2% of the total cell mass of an adult pancreas [65]. The coding genes and proteins expressed in the pancreas are described and characterized in various publications [66]. However, to the best of our knowledge, a reference list from a free-labeled MS approach analyzing the subset of proteins secreted by the islets or β-cells is not yet available. Secretome proteins have a major role in central physiological processes and are considered as a rich resource for the identification of biomarkers and drug targets. Quantitative proteomics of secreted proteins is possible by MS and has been used before [67–69], but has proven difficult and highly biased due to methodological limitations. These limitations have been partially addressed with the development of techniques such as the SPECS (the secretome protein enrichment with click sugars) that allow proteome-wide identification of secreted proteins at high-depth [70]. Further improvements can be achieved with a sophisticated *in silico* approach designated to integrate specific protein datasets already available, as presented here.

The current version of out tool does not integrate any database capable to distinguish with certainty proteins that are unconventionally secreted, which will be in an important update in the future. However, these groups of proteins could be inferred to a certain degree by first positively filtering for annotated secreted proteins and then subtracting the proteins with +SP – XXEL. Our *in* silico secretome prediction script can be adjusted to the investigation of any kind of proteomic data from any tissue or cell type.

## Conclusion

In the present study, we demonstrated how different functionalities of diverse computational tools can be used together to extract useful information in order to predict secreted proteins from proteomic data. All the programs used are open source tools freely available. Our approach reduced whole cell proteomes (HPI, MPI, and β cell) and filtered data from ISG and supernatants from potential contaminants and non-secreted proteins, predicting their respective classical secretomes.

As the analytical performance of proteomic platforms will continuously improve, resulting in better experimental description of secretomes, their output will identify new physiological processes and contribute to the identification of new therapeutic targets.

## Data Availability

Python tool created by us is freely available for download and usage at https://github.com/tatiorli/ParseSignalPeptides. The files with proteins lists (.xlxs) data used to support the findings of this study are included within the supplementary information file(s).

## Abbreviations

Asp: aspartic acid
DTT: 1,4-dithiothreitol
ECM: extracellular matrix
ER: endoplasmic reticulum
*erd2*: ER lumen protein-retaining receptor
FBS: fetal bovine serum
Glu: glutamic acid
HDEL: histidine–aspartic acid–glutamic acid–leucine
His: histidine
HPI: human pancreatic islets
IAM: iodoacetamide
ISG: insulin secretory granules
KDEL: lysine–aspartic acid–glutamic acid–leucine
LC-MS: liquid chromatography-mass spectrometry
Leu: leucine
Lys: lysine
MPI: mouse pancreatic islets
MS: mass spectrometry
MWCO: molecular weight cut-off
ppm: parts per million
PROMALS3D: PROfile Multiple Alignment with predicted Local Structures and 3D constraints
RT: room temperature
SP: signal peptide
+SP: proteins containing signal peptide
SPECS: secretome protein enrichment with click sugars
SUP: β-cell supernatant
SUP2: our own supernatant of β-cell model cells (INS-1E)
TFA: trifluoroacetic acid
UniProtKB: UniProt Knowledge
XXEL: any amino acid – any amino acid – glutamic acid – leucine (the ER-retrieval signal consensus)
+XXEL: proteins containing the XXEL sequence
-XXEL: proteins without the XXEL sequence
